# Role of bioengineering and laborers in integration of farmland resources toward to improve dimension of sustainable agriculture in China

**DOI:** 10.1080/21655979.2020.1765523

**Published:** 2020-05-20

**Authors:** Xinyou Yu, Yunqing Liu, Yiwen Wang, Xiaoli Feng, Mingzhong Tu, Jiangsheng Chen

**Affiliations:** a College of Natural Resources and Environment, Northwest A&F University, Yangling, P.R. China; b Department of Foreign Languages, Northwest A&F University, Yangling, P.R. China

**Keywords:** Labor, farmland resource, large-scale farming, biotechnology, sustainable agriculture

## Abstract

Farmland transfer is one of the essential approaches for achieving large-scale farming and its management affects productive efficiency, environment pollution and food sustainable security supply. Present study was carried out investigation based representative agricultural development area Guanzhong Plain of Shaanxi, aimed at explore the role of biotechnology and laborers in integration of farmland toward to improve sustainable agriculture in rural China by employed the profit and Tobit models evaluation. The conclusion demonstrated that labor’s and agricultural management model as main stay, intensive farming has positive effect-based economic and environmental benefits than fragmentation management, female laborers have weaker effect on farmland renting-out behavior among smallholders while male laborers were superior promoters in increasing the area of rented-in farmland and farm scale. Finally, bioengineering development and agricultural intensification management as a rational choice that has great potential value for large-scale cultivation that contributing a promising future for achieving cleaner production, environment and human health further providing huge economic and social and environmental benefits in sustainability agriculture. Additionally, government policies require intensive intervention to accelerate large-scale management and biotechnology implementation.

**Abbreviation**: *Aaflf*: Average age of female labor force; *In*com*(log)*: Log of annual household income; *Noflf*: Number of women in the labor force; *Nooaf*: Number of old adults in family; NTFs: non-transfer families; OLS: ordinary least square; *Palff*: Proportion of agricultural laborers in the female labor force; *Palmf*: Proportion of agricultural laborers in the male labor force; RIFs: rented-in families; ROFs: rented-out families; *Whhf*: Whether the household head is female

## Graphical Abstract

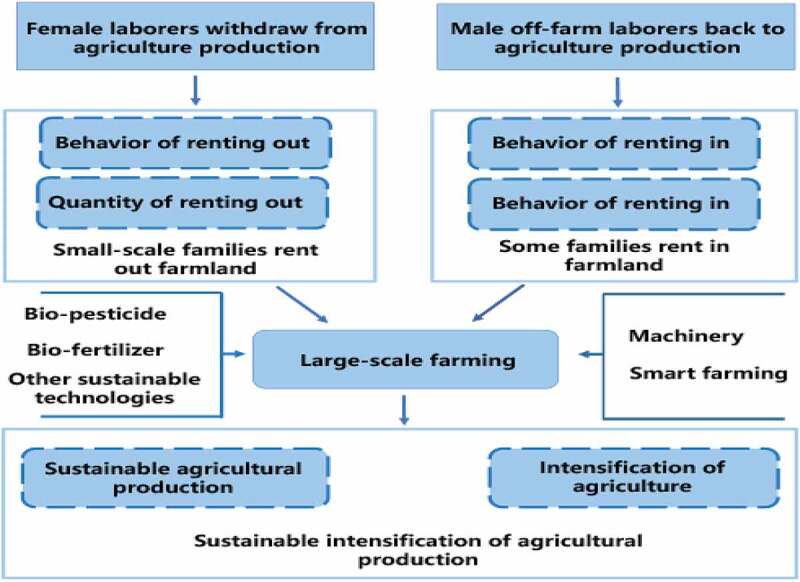

## 1. Introduction

Farmland as the basis of organism (plants, animals, and microorganisms) that was considered to be one of the important culture resources. While, extensive application of fertilizers and pesticides were greatly contributed to grain output also caused long-standing of soil and environmental pollution since the 20th century. As reported by Ministry of Ecology and Environment of the People’s Republic of China, the probability of pollution cultivated land was exceeded standards reached 19.4% in 2014 [1]. In order to improve environmental protection and agricultural product safety, it’s indispensable to develop environmentally friendly techniques and advanced effective methods for farmland management [2].

As a fundamental role in agriculture, labor’s and agricultural management model towards agricultural resources management and biotechnology play an important role for food security and sustainable agriculture. Small family farms (below 2 ha) were the main farmland management unit for a long time and contributed to the world’s food supply, which represent 84% of farms globally [3,4].China’s agriculture is typically dominated by low-efficiency smallholders and fragmented farmland [5]. However, land fragmentation of small farms caused difficulties in implementing biotechnology, which were unfavorable to the environment protection and the sustainable food supply [6,7]. Thus, agricultural intensification-based large-scale farms has become a universally adopted trend [8] that not only associated with multiple technologies (for example, agricultural mechanization and diversified use of resources [9]) and efficient management to ensure secure quantity of food [10], but also contributed to achieving cleaner production in sustainability agriculture [11,12].

The proportion of rural off-farm employment had increased quickly, as a result of rapid urbanization and industrialization since the Reform and Opening Up, numerous and rapid outflow of rural laborers to city caused the weakening of the agricultural labor capacity and waste of land resources [13,14]. In this process, male laborers are driven to migrate to nonagricultural employment [15]. The participation of women in farming management decisions was steadily increasing and agricultural feminization commonly occurring in rural China [16–18].While, it is not surprising that female-managed agricultural lands have lower productive than male-managed. However, when benefiting from competitive and efficient markets and the application of agricultural technologies and mechanization that conductive to the enhancement of efficiency [19,20]. While to stabilize the current farming group, China’s government implemented a policy in 2019 that maintains the land contract relationship with farmers for further 30 years. As one coin has two sides, this fragmented farmland becomes the cornerstone of agricultural expansion and intensive agriculture to guarantee food security in China [21,22]. Therefore, it is essential for farmland intensification to resolve land fragmentation to recovery of farmland resources in the sense of utilization efficiency and achieving agricultural expansion and large-scale farming for sustainable agriculture.

**Figure 1.**
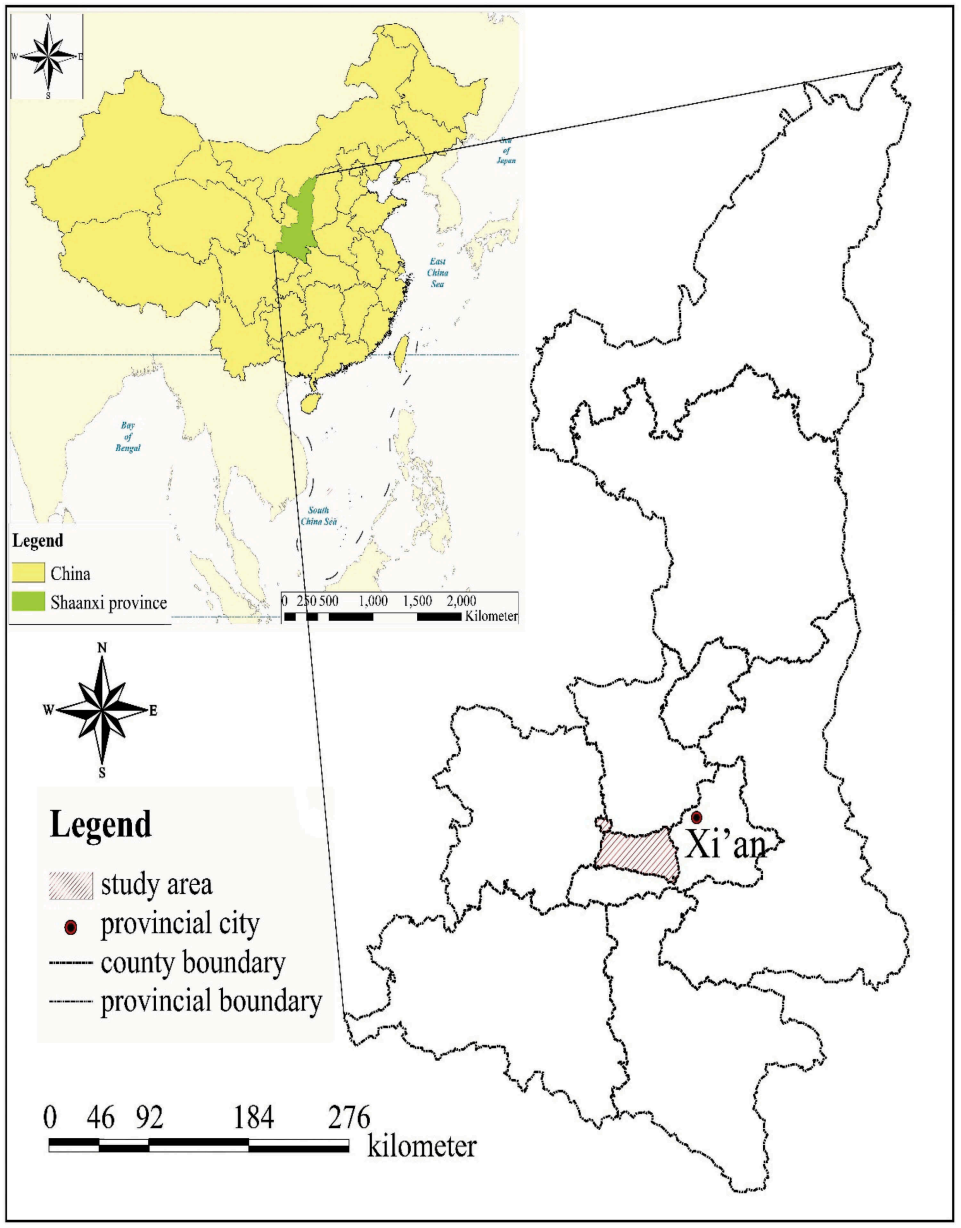
Location of study area.

The emerge of biotechnology was provided new approach for overcome those obstacles in agricultural production [23]. Such as amendment of biochar in fertilizers, fertilizers from biological waste, genetically modified crops, bio-pesticide provides valid approach to reduce environmental cost and increase sustainability of agricultural production in intensive agriculture. Organic farming is environmentally friendly and less external cost while it still facing lower yields and more difficulties in soil nutrient management which is suitable for large-scale farming (Figure 1). However, adoption of this techniques in small-scale farmers still face big challenges like availability of adequate technology information and cost of investment [24,25]. Overall, bioengineering based on genetics, cells, microbial fermentation, enzyme and bioreactor engineering, which has huge potential value integrated with large-scale cultivation, contributing a bright future for the solution of the problems facing the world, such as resources, environment and human health further providing sustainability agriculture with economic and social and environmental benefits.

Therefore, technology development and intensive management become a rational choice. However, there are no studies to discuss farmland rental participation and the extent to which families’ life quality improves through the contribution of rural laborers, which is crucial to development of large-scale farming. In order to fully excavation the potential of biotechnology and labors in farmland resource integration to achieve agricultural intensification improve the scale of sustainable agriculture development in China. This study based on investigation to determine the role and impact of laborers and biotechniques in agricultural land expansion and sustainable life quality improvement.

## 2. Materials and methods

### 2.1. Research site and survey sampling

The study area includes Yangling, Zhouzhi County and Huyi District, which are situated in the Guanzhong Plain of Shaanxi Province (Figure 1). Yangling is the national agricultural high-tech industry demonstration zone, and its farmland rental rate and level of agricultural technology application relatively high. The land rental market of Zhouzhi is developing as a traditional agricultural zone. Huyi District is close to Xian and confronting urban expansion and farmland encroachment. This study carried out in Guanzhong Plain due to it is a representative agricultural development area owing to: ① Its farmland is flat enough to achieve scale farming and mechanization; ② Has a medium-level urbanization rate and GDP in China and the major attraction of Xi'an that can be support many rural laborers engaging in off-farm employment, which means a large amount of potentially available abandoned farmland existed. ③ The area accounts for almost 48% of the total cultivated land as the main grain production area of Shaanxi Province, which means enough agricultural population and enough land to develop large-scale farming.

The farmland transfer survey was conducted in January 2018, randomly selected 48 villages and investigated approximately 12 rural households in every village, ultimately obtained 592 valid questionnaires.

### 2.2. Farmland transfer evolution mechanism

**Figure 2.**
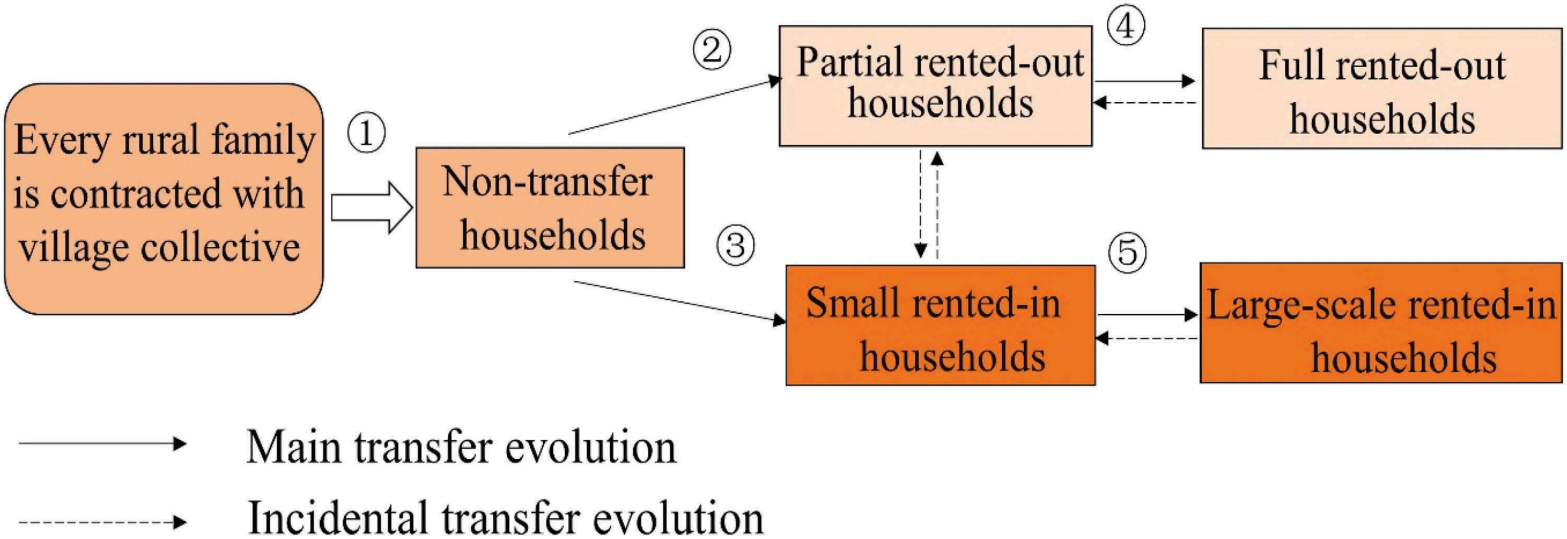
Farmland transfer evolution mechanism.

Due to the scant of farmland and famers has longer right with 30 years more in China, large-scale farming can only be achieved by a process in which millions of smallholders rent out their farmland voluntarily to a few families. This process is associated with future food security and social stability, and the mechanism is described in Figure 21. There need to be emphasized was that the assigned or contracted land is the farmland received by rural families when contracting with the village collective (shown as ① of Figure 2). Here, assigned farmland was indicated the land acquired from the village collective. At the legal level, land ownership is held by the village collectives instead of villagers that has contract and management rights over the land. Rural families can rent out or rent in the management rights for farmland during the contract period (shown in ②–⑤ in Figure 2). The concept of large-scale farming in present study was not precise size but simply a process that transfers farmland from a small and fragmented state into a desired larger and unitary state.

Based on rural families’ farmland rental choice and divided rural families into three categories: rented-out families (ROFs), rented-in families (RIFs) and non-transfer families (NTFs). ROFs was partially or entirely rented land out, while RIFs was rented land in and NTFs without land renting. Labor and land endowment were the dominant factors to effect household decision-making and transfer farmland, which is a key cornerstone to achieving life quality improvement of large-scale farming.

### 2.3. Model specification

The probit model is a widely used nonlinear regression approach in behavioral economics. And it has obvious advantages than ordinary least square (OLS) when deal with a binary (0/1) dependent variable [26]. The respondent either rent out (Y = 1) or does not (Y = 0) in the period in which the survey is taken. The household’s behavior to rent out is definitely a binary variable, and so do the behavior to rent in. That is why probit model is employed in this research. Rural households’ decision to rent land is affected by laborers’ characteristics and household characteristics, such as the number of laborers, household income, political status, and land endowment. Present study based on these variables and performed assessment by probit model, aimed at identified each variable’s contribution to a household’s decision by:

(1) $$P(y_{i}^{\;}=1)=\alpha_{1}^{\;}+\beta_{1}^{\;}x_{1}^{\;}+\theta_{1}^{\;}z_{1}^{\;}+\mu_{i}^{\;}$$

where *y_i_* indicates the farmland rental decision of household *i*. The independent variable is a dummy variable in Equation (1), *y_i_ *= 1 represents household *i* rent out or rent in land; otherwise, *y_i_*is 0. **X***_1 _*is a vector of female labor characteristics that may influence household behavior on the land market, **Z***_1 _*is a vector of household characteristic variables, and *μ_i_* is a stochastic error term.

Next, employ the Tobit model to estimate the amount of rental farmland by:

$$y_{i}^{*}=a_{1}^{\;}+b_{1}^{\;}x_{1}^{\;}+c_{1}^{\;}z_{1}^{\;}+\epsilon_{i}^{\;}$$

(2) $$y_{i}^{\;}=\left\{\begin{array}{c} y_{i}^{\;},ify_{i}^{*}>0\\ 0,ify_{i}^{*}\leq 0 \end{array}\right.$$

where $y_{i}^{*}$ is latent variable, *y_i_* indicates rented-in area or the proportion of rented-out land of household *i*. **X***_1_* is a vector of female labor characteristics, **Z***_1_* is a vector of household background variables, and *ϵ_i_* is a stochastic error term. Additionally, evaluate the rural families’ quality of life under farmland transfer.

### 2.4. Statistical analysis

All the results of the empirical analysis were estimated in SAS 9.4 software package (2014, V. 9.4, SAS Institute Inc., Cary, NC, USA.). The tolerances of all variables were higher than 0.4 that means no serious multicollinearity among these regression models [27].

## 3. Results and discussion

### 3.1. Descriptive statistics of variables

Relevant variables in this research described the women’s characteristics, household characteristics and farmland rental decisions as present in Table 1. It must be explained that the definition of the labor force has additional constraints in this study: labors over 18 years of age are capable of working and the member of the labor is not student, farmers over 60 years old still working also consider as a the labor force due to the participation of aged over 60 years old in agricultural production in rural areas is common.

Table 1 reveals that farming is the major career for rural female labor force with the mean value of *Palff* (0.611) higher than *Palmf* (0.543). Thus, agricultural feminization is occurring. Female of small-scale farms pay more attention to the convenient technologies for insect control and labor saving [27]. The mean *Whhf* is 0.047 that implies approximately 5% of household heads are female and male are the mainstream form of rural families. Additionally, the average level of education in the female labor force was 8.083 years, and the average age of was 44.379. Furthermore, the assigned land for a rural family averaged 0.316 ha, which is lower than China Labor Force Dynamics Survey in 2014 (0.381 ha) [14]. The desire of rural families to rent land was weaker that only 38.7% of households choose to transfer land, over 60% of households not rent farmland. Therefore, it is quite urgent to search for potential ROFs in these NTFs, while it will be a long process to achieve large-scale farming.

**Table 1.** General descriptive statistics of some variables for the whole sample.

Variable	Description	Mean	SD
Women’s variables			
*Noflf*	Number of women in the labor force	1.532	0.670
*Palff*	Proportion of agricultural laborers in the female labor force	0.611	0.381
*Aeflf*	Average years educated of female labor force	8.083	3.566
*Aaflf*	Average age of female labor force	44.379	12.564
*Whhf*	Whether the household head is female (female = 1; male = 0)	0.047	0.212
*Palmf*	Proportion of agricultural laborers in the male labor force	0.543	0.351
Household characteristics			
*Nolab*	Number of laborers	3.358	1.107
*Numoc*	Number of children (age<18)	0.782	0.856
*Nooaf*	Number of old adults in family (age≥60)	0.956	0.913
*Heada*	Age of household head	55.242	9.904
*Heade*	Years educated of household head	8.465	3.092
*Offic*	Whether one of the family members is a rural official (yes = 1; no = 0)	0.063	0.242
*Poain*	Proportion of agricultural income	0.251	0.260
*Incom*	Annual household income (thousand USD) ^a^	18.636	42.216
*Aipla*	Average annual income per laborer (thousand USD) ^a^	5.871	16.918
*Lsati*	Livelihood satisfaction, which has five options: extremely dissatisfied, dissatisfied, general, satisfied and very satisfied, respectively ranked from 1 to 5.	2.481	0.929
Land characteristics			
*Aopla*	Area of planting (ha)^b^	0.568	2.155
*Aland*	Area of assigned land (ha)^b^	0.316	0.137
*Aifph*	Annual input of fertilizers and pesticides per hectare (thousand USD per hectare)	1.985	2.371
*Aroaw*	Whether adopted recycling of agricultural waste (yes = 1; no = 0)	0.277	0.448
Dependent variables			
*Renou*	Whether family rented out its farmland (yes = 1; no = 0)	0.228	0.420
*Prold*	Proportion of rented-out land in assigned land	0.177	0.352
*Pagin*	Proportion of agricultural income in total income	0.251	0.260
*Renin*	Whether family chose to rent in farmland (yes = 1; no = 0)	0.159	0.366
*Arild*	Area of rented-in land (ha)	0.305	2.139
Observations		592	

^a^100 USD = 661.74 CNY (Annual data of exchange rate from National Bureau of Statistics of China in 2018 (available at http://www.stats.gov.cn/tjsj/ndsj/2019/indexeh.htm)).

^b^1 ha = 15mu.

### 3.2 The impact of the labor force on farmland rental decisions

According to Eq. (1) evaluated the variables’ contributions to household decision for land transfer, through samples of ROF and NTF to regress the model Renou, which estimates the possibility that rural families will rent out their farmland. The results are shown in Table 2, families with fewer female laborers are more likely to rent out land, it is observed that *Palff* and *Palmf* were negative and statistically significant at the 0.01 level. The decreased proportion of agricultural labor force implies that the agricultural labor force turned to work in off-farm sectors, driven by the higher economic return of off-farm employment. Obviously, the remaining laborers cannot manage the original area of the land and thus more likely to rent out.

Meanwhile, the absolute value of *Palff* is higher than *Palmf* that suggested that the female labor force is more influential than the male labor force in affecting farmland transfer out. This might be caused by male farmers find off-farm employment in cities, but the local employment of women is usually unstable due to incomplete development of the rural labor market [28], female labor will take over the land and renting-out behavior has less probability of occurring. Most young rural women and men prefer working in cities, and they are usually off-farm employees. Those manage farmland labor are middle-aged and elderly women because their age, education and professional skills suppressed to find stable employment in cities. Therefore, the lower value of variable *Aaflf* still implies that the local off-farm employment of laborers may increase the probability of renting-out land [29,30], it is critical to create more local off-farm employment farmers to facilitated the farmland rental process and achieve large-scale farming. The variable *Whhf* indicated that female household heads prefer to rent out land when they manage farmland in line with [18,31].

Regarding to the model Renin estimates the possibility that rural families will rent in farmland by the samples of RIF and NTF. The results show the variables *Palff* and *Palmf* are statistically significant. It suggests that a higher proportion of agricultural labor in the female or male labor force means a higher possibility of renting in, the influence of the female labor force on renting farmland is consistent with males. Additionally, *Noflf* has opposing significance and signs in models Renou and Renin that implies the families with fewer female laborers are more likely to rent out and the number of female laborers will not affect the probability of renting in. This finding also supports the previous opinion that most remaining female laborers are engaged in farm work and the behavior of renting in is dominated by males, since *Noflf* can be significant of female laborers would like to rent in [32,33].The parameter of *Nooaf* suggests that families with fewer old adults will have higher probability of renting in. Variable *Incom(log)* has the opposite sign in models Renin and Renou that suggests low-income smallholders prefer to rent out and high-income families would like to rent in due to more abundant property and stronger ability to manage risk.

### 3.3 The impact of the labor force on farmland rental quantity

According to Eq. (2) identified the contributions of each variable to quantity of farmland that households decide to rent out or rent in. The results are shown in Table 3 from the samples of ROF and NTF to regress the model Prold, the dependent variable of *Prold* was the proportion of rented-out land in assigned land (0–1). As concluded the variables *Palff* and *Palmf* were significant and had negative signs, and the absolute value of *Palff* was larger, which indicated the female labor force has a slightly more powerful effect than the male labor force on the proportion of rented-out land. As the result of probit model in Table 2, compared with male farmers engaged in off-farm employment, the probability of renting out will be larger when female farmers engage in off-farm employment. However, this model shows that neither a large nor the full portion of land will be rented out if female farmers turn to local off-farm employment or migrate out that because older adults sometimes take over the land. Although elderly women may be limited on physical and attention, while they can manage more land due to the purchase of rural agricultural services and use of agricultural machinery. When elderly men cannot or are unwilling to cultivate the remaining land, it can be rented out entirety.

**Table 2.** The impact of the female labor force on farmland rental decisions.

Variable	Renou		Renin	
	Coef.	SE	Coef.	SE
Intercept	1.799***	0.660	−6.285***	1.151
*Noflf*	−0.530***	0.135	0.052	0.187
*Palff*	−1.646***	0.249	1.100***	0.403
*Aeflf*	0.018	0.025	0.015	0.031
*Aaflf*	0.015**	0.007	0.024	0.015
*Whhf*	0.564*	0.326	0.147	0.458
*Palmf*	−1.374***	0.265	1.142***	0.311
*Numoc*	0.068	0.089	0.069	0.098
*Nooaf*	0.006	0.093	−0.261**	0.106
*Heada*	−0.011	0.009	−0.016	0.011
*Heade*	0.010	0.026	0.038	0.030
*Offic*	0.050	0.322	0.098	0.330
*Incom(log)*	−0.338**	0.141	1.442***	0.201
*Aland*	1.105*	0.579	−1.717***	0.637
AIC	429.143		358.527	
Log Likelihood	−200.571		−165.263	
Observation	498		457	

The symbols*,**, *** indicate statistical significance at the 10, 5, and 1% levels, respectively.

The significance and sign of *Aaflf* in model Prold also supports the opinion that female laborers with higher education and human capital will prefer to rented out. The negative signs of *Heada* are rational for younger household heads who prefer to work in off-farm sectors [34]. The samples of RIF and NTF to regress the model Arild that is the area of rented-in land. In this model, observed that the absolute value of *Palmf* was larger than *Palff,* which indicated males managing farmland will rent in more land than female farmers. This may be because of female laborers are usually less powerful in the decision-making than males; male laborers hold more household resources and more household productive assets will be used as input when they turn to farming; male laborers are more eager to rent in farmland that caused the main promoter for increasing the area of rented-in farmland and farm scale. But male’s pursuing higher yields may cause heavy use of pesticides and fertilizer that have negative impact on agricultural sustainability [28].

The significance and sign of *Noflf* was similar in Tables 3 and 2, families with fewer female laborers are more likely to rent out and rent more land, while the number of female laborers will not affect probability and quantity of renting in. The sight significance of *Aaflf* may result from higher education and the stable off-farm employment of women, increasing the confidence of males in their investment in agriculture, which also implies the risk-averse position of female laborers in their families [32]. Furthermore, from the estimated parameter of variable *Nooaf* and *Incom(log)* also obtained that older adults have a negative impact on the decision and quantity of renting in and rural families with relatively high farm income are better able to expand their farm size and gain greater economies of scale [34,35].

**Table 3.** The impact of the female labor force on farmland rental quantity.

Variable	Prold		Arild	
	Coef.	SE	Coef.	SE
Intercept	3.981***	0.904	−25.126***	4.217
*Noflf*	−0.602***	0.179	0.374	0.674
*Palff*	−2.452***	0.389	2.922**	1.360
*Aeflf*	0.018	0.031	0.072	0.120
*Aaflf*	0.019**	0.009	0.103*	0.056
*Whhf*	0.481	0.406	−0.621	1.699
*Palmf*	−2.382***	0.414	4.426***	1.097
*Numoc*	0.026	0.114	−0.061	0.373
*Nooaf*	0.002	0.120	−0.907**	0.397
*Heada*	−0.025**	0.011	−0.040	0.042
*Heade*	0.017	0.032	0.089	0.111
*Offic*	0.046	0.406	−0.167	1.128
*Incom(log)*	−0.639***	0.190	4.992***	0.456
*Aland*	1.281*	0.769	−2.769	2.245
AIC	547.239		721.014	
Log Likelihood	−258.619		−345.507	
Observation	498		457	

The symbols *, **, *** indicate statistical significance at the 0.1, 0.05, and 0.01 levels, respectively. AIC: Akaike information criterion.

**Table 4.** Differences of livelihood status and pro-environmental behaviors among three classes of agricultural families.

Variable	NTFs	Partial ROFs	RIFs	F Value
*Pallf*	0.693 (0.316)	0.570 (0.404) **	0.764 (0.312)	8.080(0.000) **
*Palmf*	0.618 (0.308)	0.531(0.287)	0.660 (0.313)	0.530(0.589)
*Poain*	0.253 (0.215)	0.081 (0.102) **	0.539 (0.293) **	19.370(0.000) **
*Incom*	13.129 (7.837)	13.236 (7.176)	47.469 (100.021) **	8.850(0.000) **
*Aipla*	3.784 (1.746)	3.847 (2.077)	16.236 (40.817) **	5.740(0.003) **
*Lsati*	2.515 (0.935)	2.710 (0.857)	2.213 (0.949) **	0.750(0.473)
*Aopla*	0.322 (0.140)	0.175 (0.111)	2.220 (5.105) **	8.590(0.000) **
*Aifph*	2.179(1.929)	1.734(1.867)	2.945(3.837) **	3.040(0.048) **
*Aroaw*	0.281(0.450)	0.435(0.500) **	0.362(0.483)	2.270(0.105)
Observation	363	62	94	

The symbol ** indicates statistical significance at the 0.05 level. When performing multiple comparisons, some variables passed Levene’s homogeneity of variance test at the 0.05 level except some variables. Those test-passed variables were tested by Dunnett’s two-tailed t-test, in which NTFs was the control group. For those variables that failed in Levene’s homogeneity of variance tests, the Games-Howell method was employed to compare, but only the comparison with NTFs was reported to be consistent with past variables.

### 3.4. An analysis of life quality improvement based on the farmland rental decisions of rural families

Based on households that rent out their farmland in full form with different employment status and lifestyle than those rent out part of farmland, present study divided ROF into Partial ROF and Full ROF. Then, compared and evaluated the lifestyle and life quality of four classes by NTF, Partial NOF, Full ROF and RIF, while NTFs as control. From the rented-in dimension, there is no doubt that RIFs have the highest area of planting (almost 2.22 ha) which is 6.9 times than NTFs. Meanwhile, average total income and income per laborer are also the highest (47.469 thousand USD). If the total income is multiplied by the proportion of agri-income, it is calculated that the farming revenue of RIFs is 7.7 times than NTFs, which implies the positive scale effect of farmland and benefit from renting additional. RIFs have the highest productivity that revealed enlarge farm size and achieve economies of scale and will also benefit most from renting additional land [36–38], larger standard deviation (100.021, 40.817 and 0.949) also implies that some RIFs fail to pursue income and welfare improvement. However, RIFs are the lowest livelihood satisfaction that may imply greater pressure due to large amount of fixed asset investment request stable and maximum output, as well as unstable profit is owing to the fluctuation of agricultural production market.

Additionally, NTFs might be attracted more concerns due to they have a similar income and livelihood satisfaction as the Partial ROFs and Full ROFs as well as also considered as potential customers for large scale farming [39]. China’s government implemented many policies such as anti-poverty projects and other policies to benefit farming, which increase the incomes and quality-of-life for low-income group that most was belong to NTFs [40]. Those policies also play essential role in keeping small households from renting-out their contracted farmland. Overall, the positive or negative livelihood status of lessees, lessors and farm quitters as well as government policy will influence potential farmland transfer behavior.

### 3.5. The adoption of pro-environmental behavior

Large-scale farming is the solution to guarantee quantity and quality safety of food in the future, but the current situation of fragmented agriculture will inevitably last for a long time without the motivated of relevant policies. Thus, the agricultural sustainability and agricultural pollution reduction mainly depend on pro-environmental practices of farmers at present. While the distinct attitudes of pro-environmental practices in families with difference livelihood strategies was investigated and present in Table 4, the differences of pro-environmental practices which contain input of fertilizers and pesticides, and recycling of agricultural waste like animal manure and crop straw.

Obviously concluded that 43.5% of the Partial ROFs adopt the recycling of agricultural waste which is the most adoption and lowest invest in fertilizers and pesticides. This result seems to reveal that the Partial ROFs were more care about agricultural product quality and environmental risk instead of yield, but the true reason was complicated. Firstly, the crop was main source of family food for Partial ROFs and the quality is related to themselves health, high proportion of staple food ration will reduce the application of pesticides [41,42]. Besides, the left-behind family members have sufficient capital to adopt labor-cost and pro-environmental practices due to the remittances from off-farm laborers [43,44]. This result is opposite with [41] that reported off-farm employment has positive marginal effect on input of fertilizers and pesticides in apple farming households. Moreover, female laborers are more powerful in agricultural production than males in Partial ROFs, which caused the high adoption of recycling of agricultural waste and low invest of fertilizers and pesticides could be outcomes of female preference. Additionally, RIFs has the highest income while large amount of fertilizers and pesticides were applied to avoid negative effect of insufficient fertility, diseases and pests driven by high yields, but adopt less pro-environmental practices that indicated RIFs’ main aims at yield and income. Because of male laborers dominate farmland renting in and farm management in RIFs and yield and productivity were dominate targets for RIFs life quality improvement by maximizing agricultural income [45,46].

**Figure 3.**
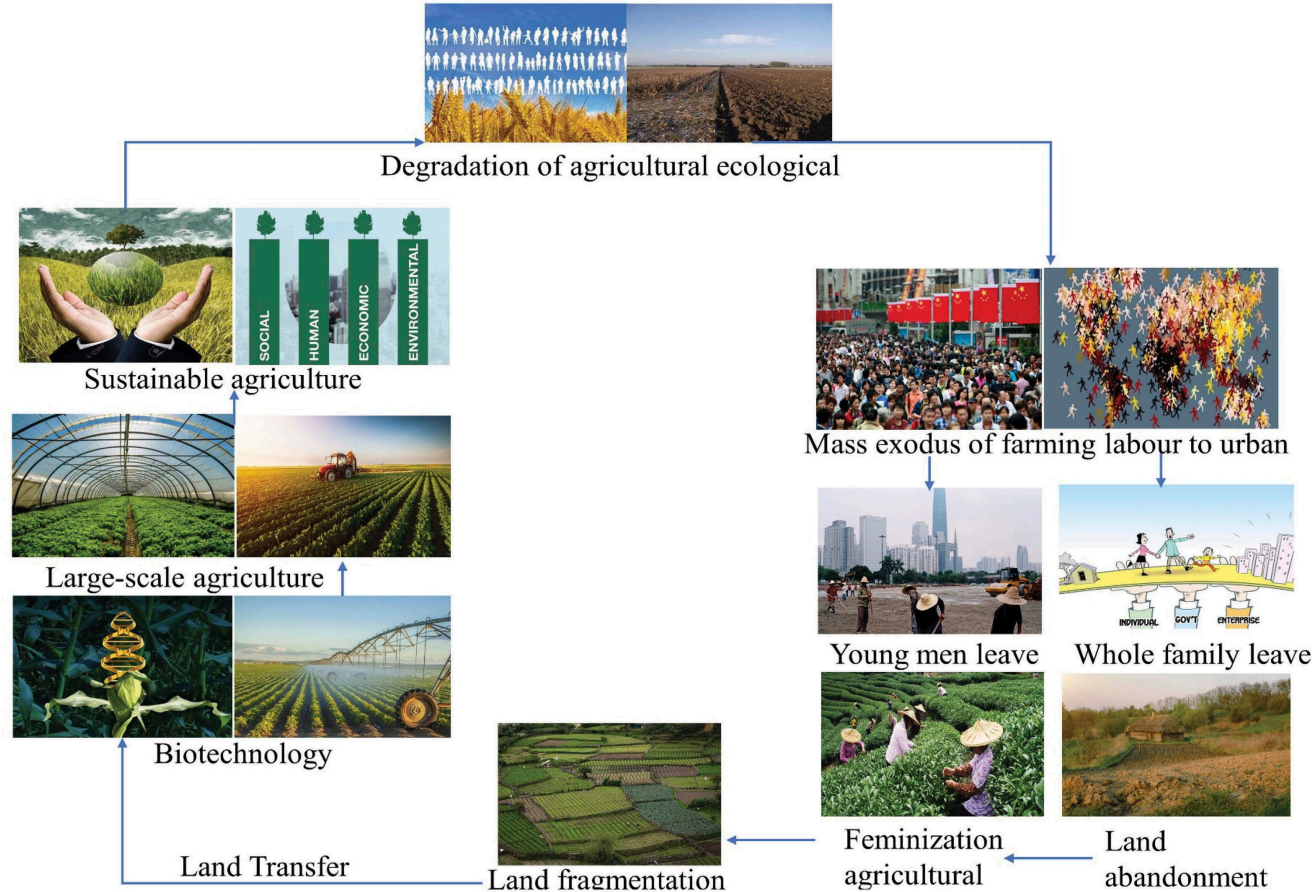
Farm land status and trend in rural China.

**Figure 4.**
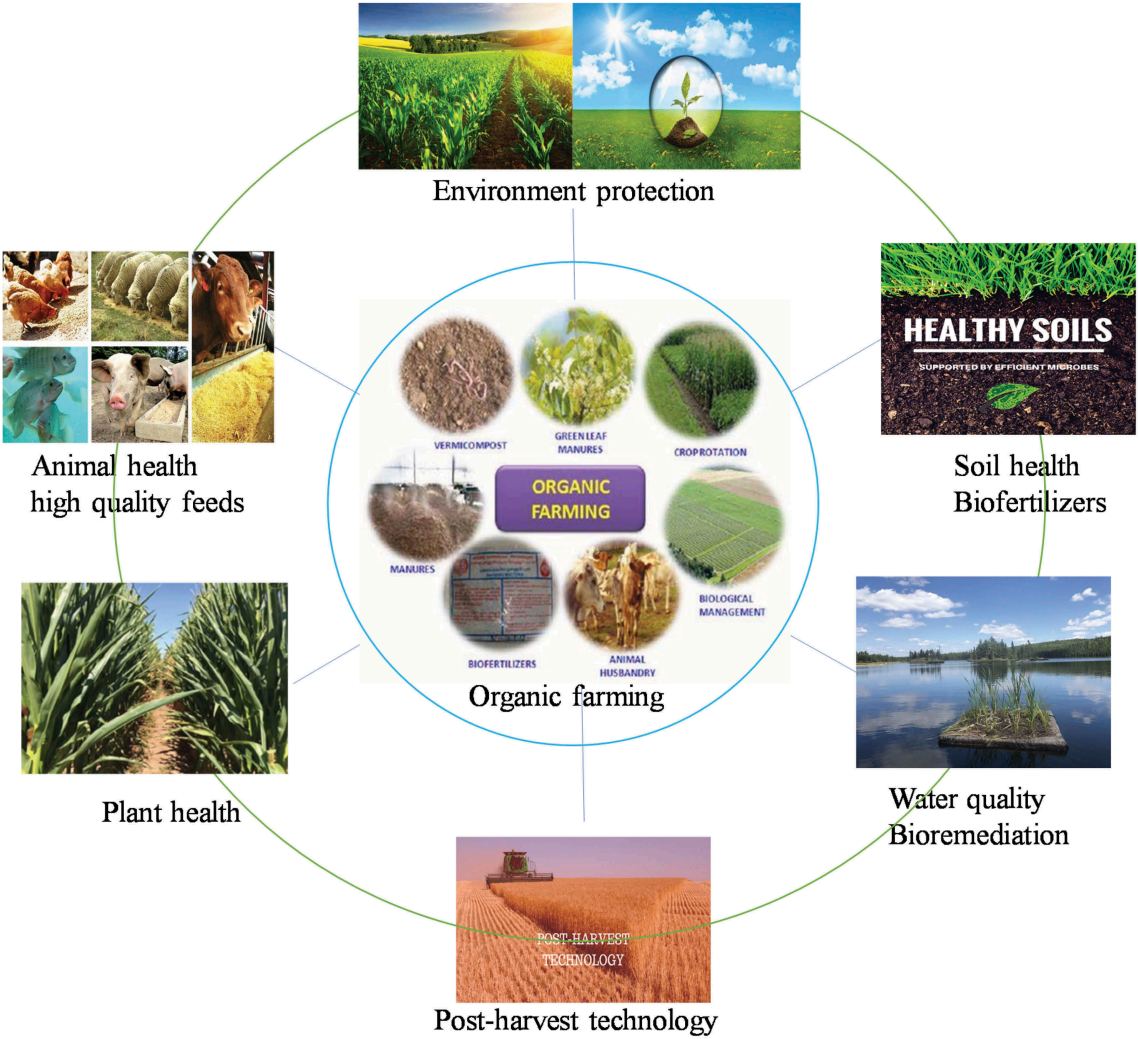
Benefit of organic agriculture.

**Figure 5.**
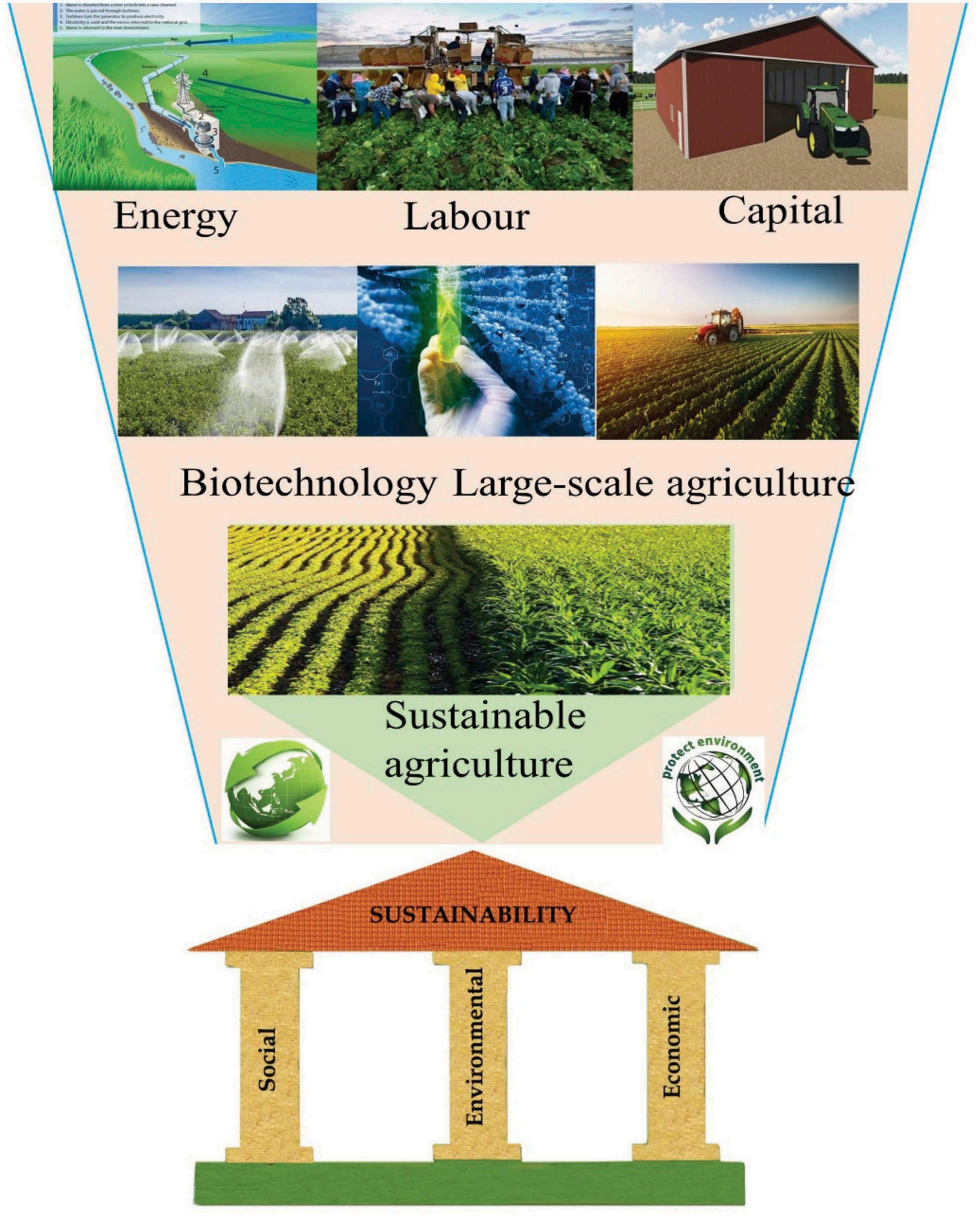
Farmland potential in sustainable agriculture.

In aspect of China current farm land condition (Figure 3), single and simple technique performed with fertilizer mainly external input by chemical fertilizer and weeding usually through herbicides with tend of feminization agricultural. Although the direct benefits of these chemical agents are indeed impressive, while they will be caused long-term harmful effects on soil and other non-target organisms that causing great potential damage to the ecosystem [47,48]. Organic agriculture approach is necessarily ecologically sustainable to improve soil quality, biodiversity and productivity, and can provide output growth that is compatible with population growth requirement and reduce time and cost (Figure 4) [31,49,50]. Thus, diversify agricultural production system-based energy, labor and capital was indispensable developed to achieving sustainability with maximize the efficiency of agricultural resources while minimize environmental risks (water, atmosphere, biodiversity, and renewable energy sources). Therefore, regarding to energy, labor and capital, the use of biotechnology and intensive large-scale management is the general trend for ecological and economic eco-friendly sustainable agriculture in China (Figure 5), which not only reduces the cost of laborers in integration of farmland resources but also improves dimension of sustainable agriculture in developing countries like China.

## 4. Conclusion and future prospect

The current investigation fully proves the positive effects of agricultural intensification management and environmentally friendly technologies was a promising choice for land resources, environment and human health, such as organic agriculture and biological control service to increase yield and sustainable agricultural production. Additionally, male laborers are the main promoters in increasing the area of rented-in farmland and female members of participating farms has more powerful in decision-making around agricultural production and farmland transfer.

Based on the above finding, the following suggestions are proposed:

- Increases stimulus policy to expand the implementation of organic agriculture under mushrooming of urbanization and population requirement.
- Enhancement government subsidy and services to organize trainings of environmentally friendly biotechnology products in order to promote adoption of biotechnologies.
- Encourage intensive implementation, expand agricultural enterprise scale and attract more investors by increase the subsidy connected with farmland transfer.
- Strengthen intensive farm operators’ awareness of circular agriculture to ensure sustainable development of agriculture, improve diversify agricultural production system contributing to the farmland market and agricultural production market.
